# Supplementary material for: Molecular and evolutionary basis of O-antigenic polysaccharide-driven phage sensitivity in environmental pseudomonads
Source: Microbiol Spectr. 2023 Oct 6;11(6):e02049-23. doi: 10.1128/spectrum.02049-23 (PMC10715155; doi:10.1128/spectrum.02049-23)
Supplement: Supplementary information — Contain all supplementary figures and tables. [file spectrum.02049-23-s0002.pdf]

SUPPLEMENTARY INFORMATION

Title: Molecular and evolutionary basis of O-antigenic polysaccharide driven phage sensitivity in environmental pseudomonads

Running title: O-antigen-mediated phage and *Pseudomonas* interaction

Jordan Vacheron[1#], Clara M. Heiman[1], Julian R. Garneau[1], Peter Kupferschmied[1†], Ronnie de Jonge[2], Daniel Garrido-Sanz[1], Christoph Keel[1#]

[1]: Department of Fundamental Microbiology, University of Lausanne, Lausanne, Switzerland.
[2]: Plant-Microbe Interactions, Department of Biology, Science4Life, Utrecht University, Padualaan 8, Utrecht, 3584 CH, The Netherlands.

[†]: Present address: Peter Kupferschmied, Federal Office for Agriculture, Swiss Federal Plant Protection Service, Bern, Switzerland

[#]**Correspondence**: Christoph Keel, christoph.keel@unil.ch, Jordan Vacheron, jordan.vacheron@unil.ch.

Content:

Supplementary figures

Fig. S1. Distribution of the transposon insertions across the genome of *Pseudomonas protegens* CHA0.
Fig. S2. Biological reproducibility of the Tn-seq results.
Fig. S3. Clusters of orthologous groups (COGs) classifications of the genes potentially involved in phage resistance according to the multiplicity of infection (MOI) conditions.
Fig. S4. Comparison of the Tn-seq data for the different phage concentrations tested.
Fig. S5. Impact of multiple and individual gene deletions within the OBC3 gene cluster of *Pseudomonas protegens* CHA0 on its phage susceptibility.
Fig. S6. Prediction and protein structure comparison of the putative pectate lyase encoded in the genome of the phage ΦGP100.
Fig. S7. Different sensitivity patterns displayed by *Pseudomonas protegens* subgroup strains that harbor the OBC3 gene cluster.
Fig. S8. The OBC3 gene cluster is located within a potential genomic island.
Fig. S9. Horizontal gene transfer signature detected within the different orthologous genomic regions of OBC3 in bacterial genomes.
Fig. S10. Maximum likelihood trees of the amino acid sequences of the corresponding genes conserved within the OBC3 gene cluster detected in bacterial genomes.

Supplementary tables

Table S1. Plasmids used in this study.
Table S2. Tn-seq characteristics.
Table S3: Phage genomes used in this study.
Table S4. Update of the phage GP100 annotation.
Table S5. Bacterial strains used in this study.
Table S6. Occurrence of inverted repeats sequences inside the OBC3 gene cluster of *Pseudomonas protegens* CHA0.
Table S7. *Pseudomonas protegens* CHA0 derivatives and *Escherichia coli* strain used in this study.
Table S8. Primers used in this study.

References

Supplementary figures

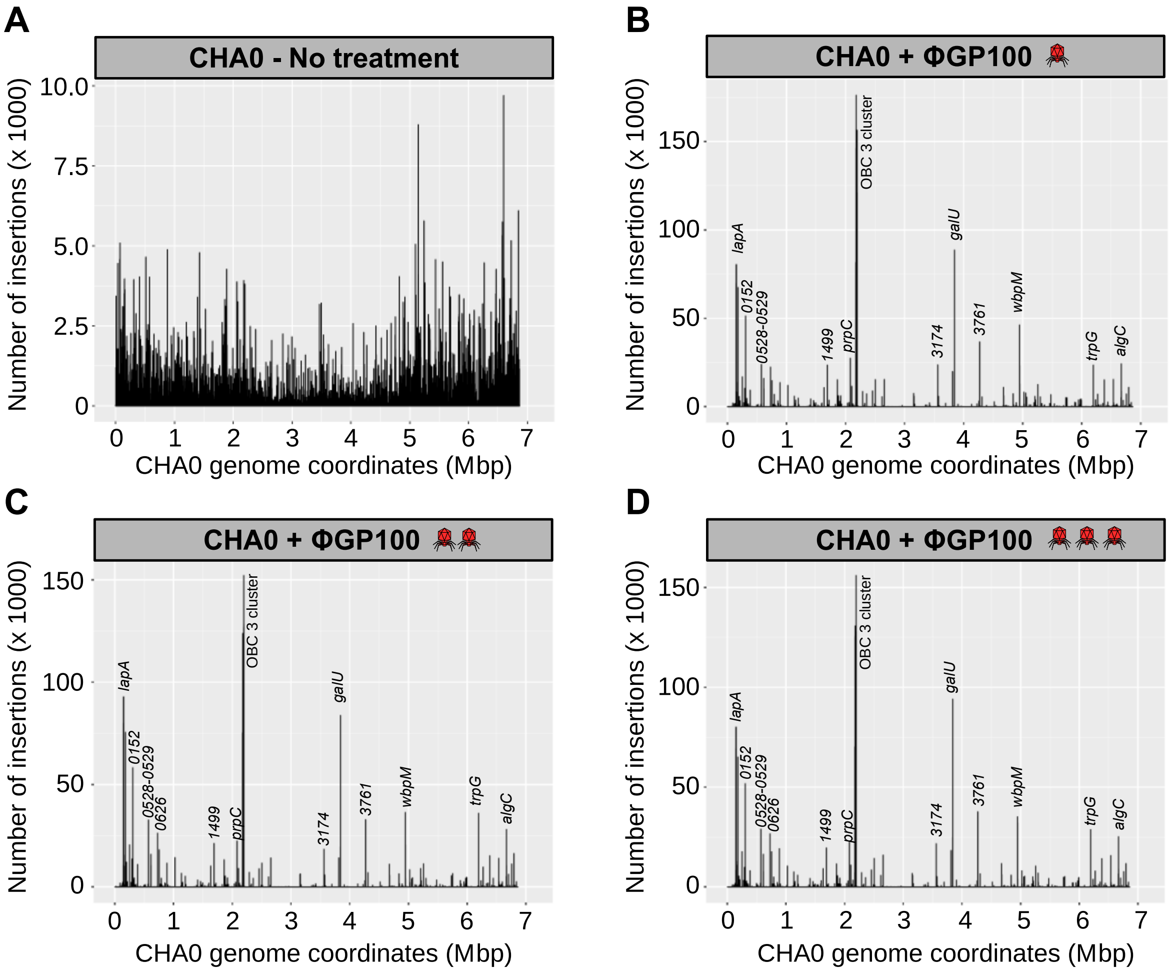

Fig. S1. Distribution of the transposon insertions across the genome of *Pseudomonas protegens* CHA0. Transposon insertions in the non-treated condition (**A**) and in presence of the phage ΦGP100 with a multiplicity of infection (MOI) of 1 (**B**), 10 (**C**) and 100 (**D**), indicated with cartoons of one, two or three red phages, respectively.

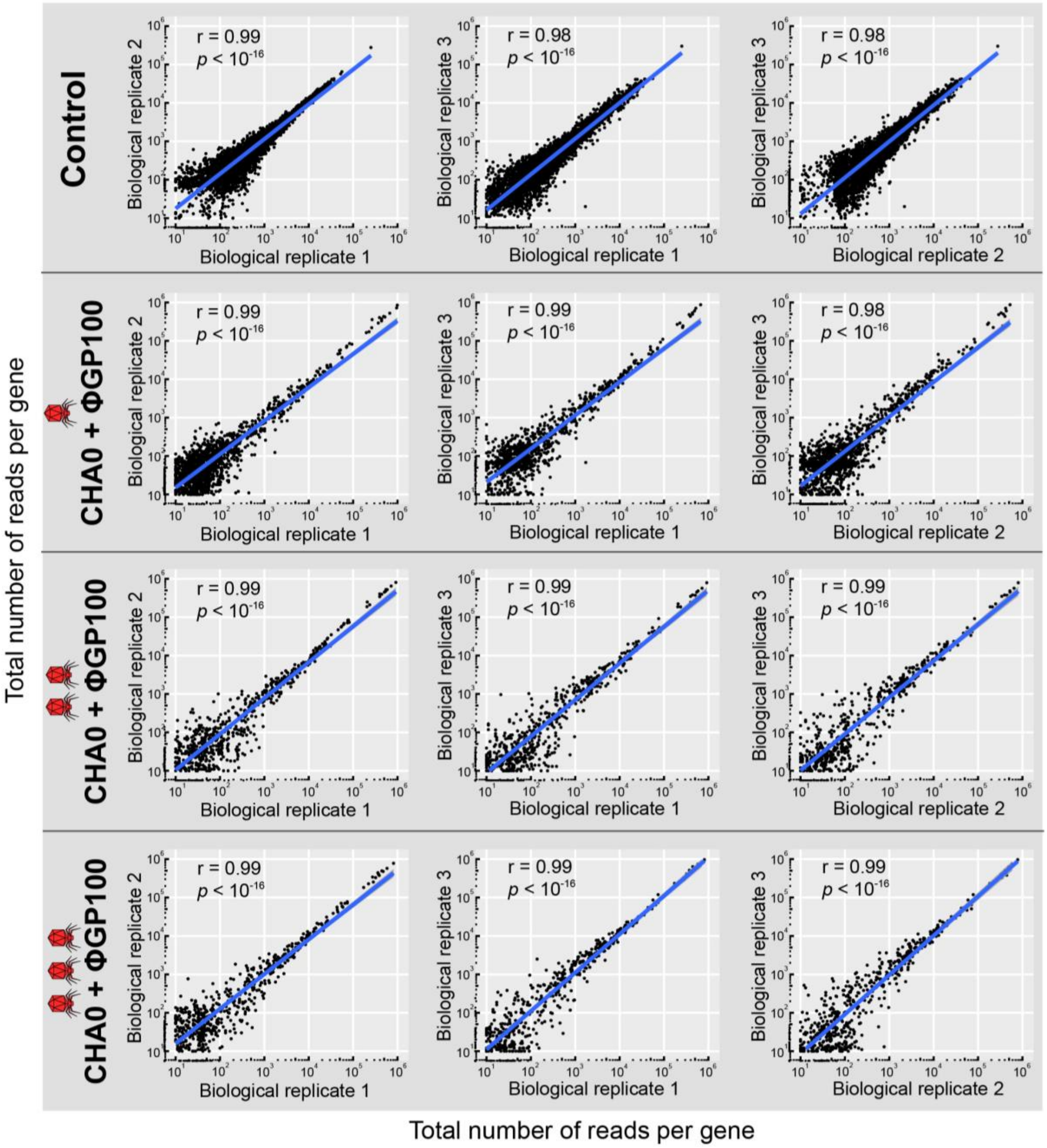

Fig. S2. Biological reproducibility of the Tn-seq results. Axes represent the total number of reads per gene for the respective biological replicates. Three independent biological replicates were compared for each condition. The different multiplicity of infection (MOI) conditions are represented with the red phage cartoons. One phage, MOI of 1; two phages, MOI of 10; three phages, MOI of 100. Control, no phage treatment. Correlation coefficients were calculated using Pearson correlation test.

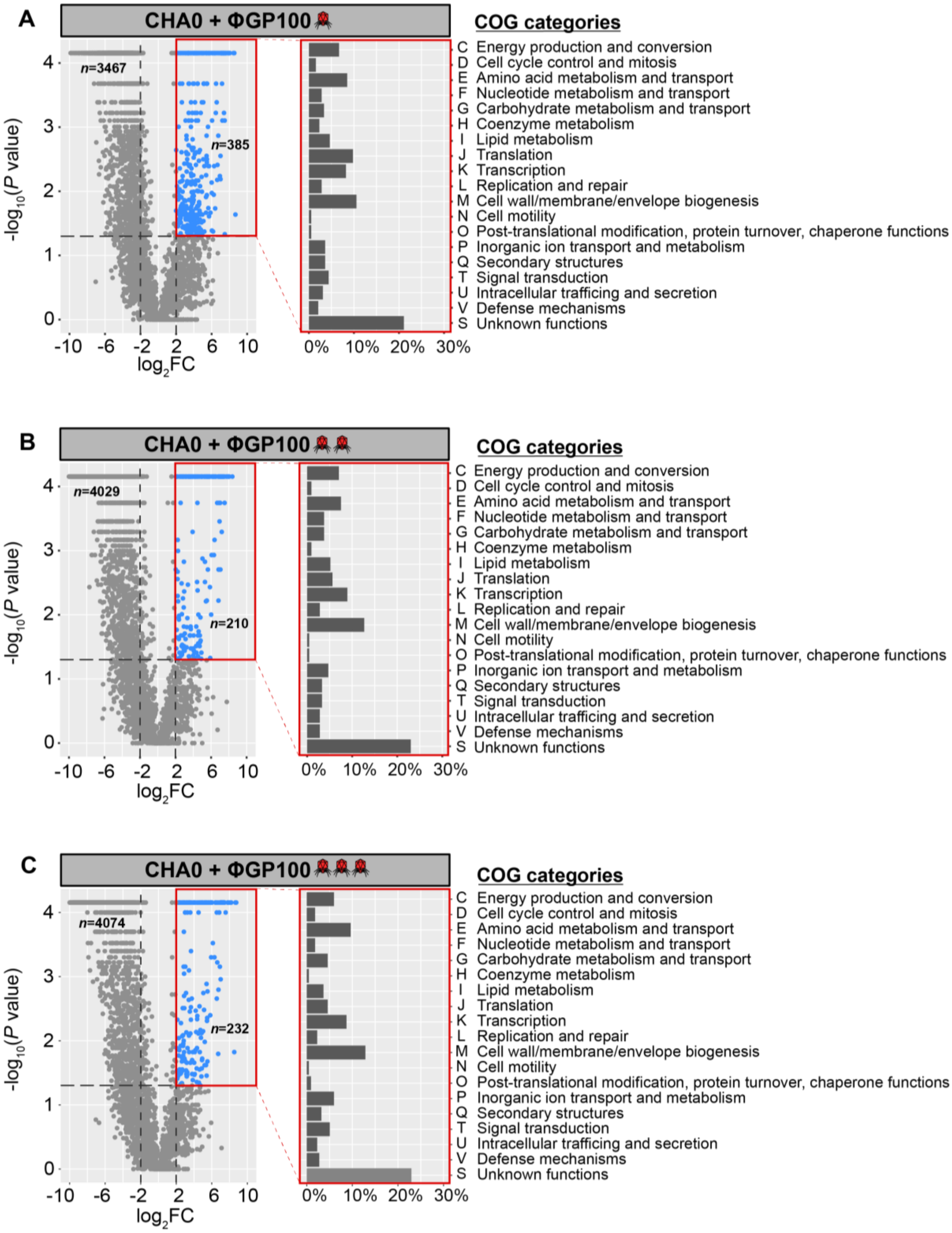

Fig. S3. Clusters of orthologous groups (COGs) classifications of the genes potentially involved in phage resistance according to the multiplicity of infection (MOI) conditions. Red phage cartoons: one phage, MOI of 1 (**A**); two phages, MOI of 10 (**B**); three phages, MOI of 100 (**C**). The blue dots correspond to genes presenting a log2FC > 2 and $p < 0.05$.

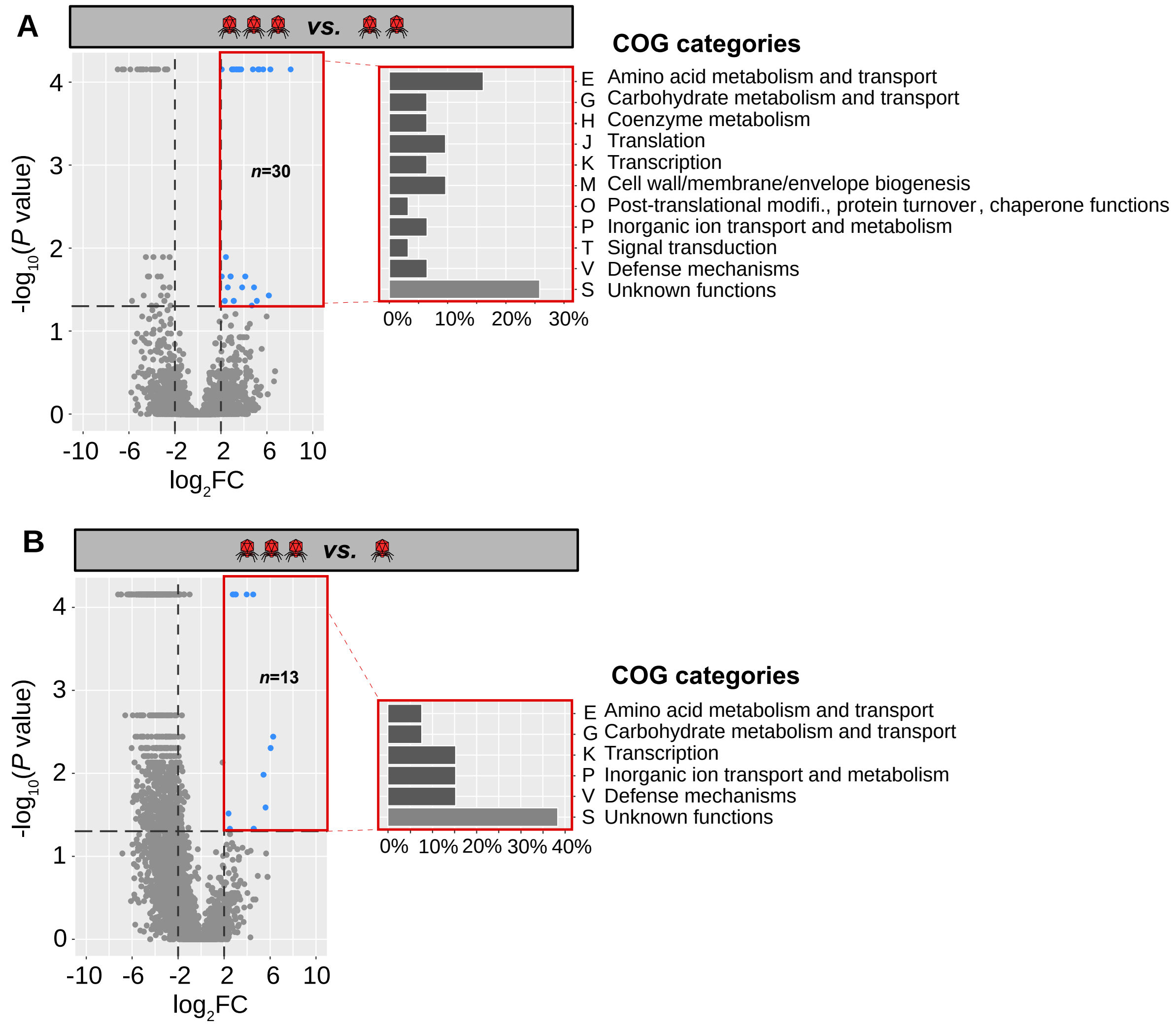

Fig. S4. Comparison of the Tn-seq data for the different phage concentrations tested. (**A**) Multiplicity of infection (MOI) of 100 vs. MOI of 10 and (**B**) MOI of 100 vs. MOI of 1. Red phage cartoons: one phage, MOI of 1; two phages, MOI of 10; three phages, MOI of 100. The genes potentially required for phage resistance are presented with blue dots (log2FC > 2 and $p < 0.05$) and classified according to the clusters of orthologous groups (COGs) they belong to.

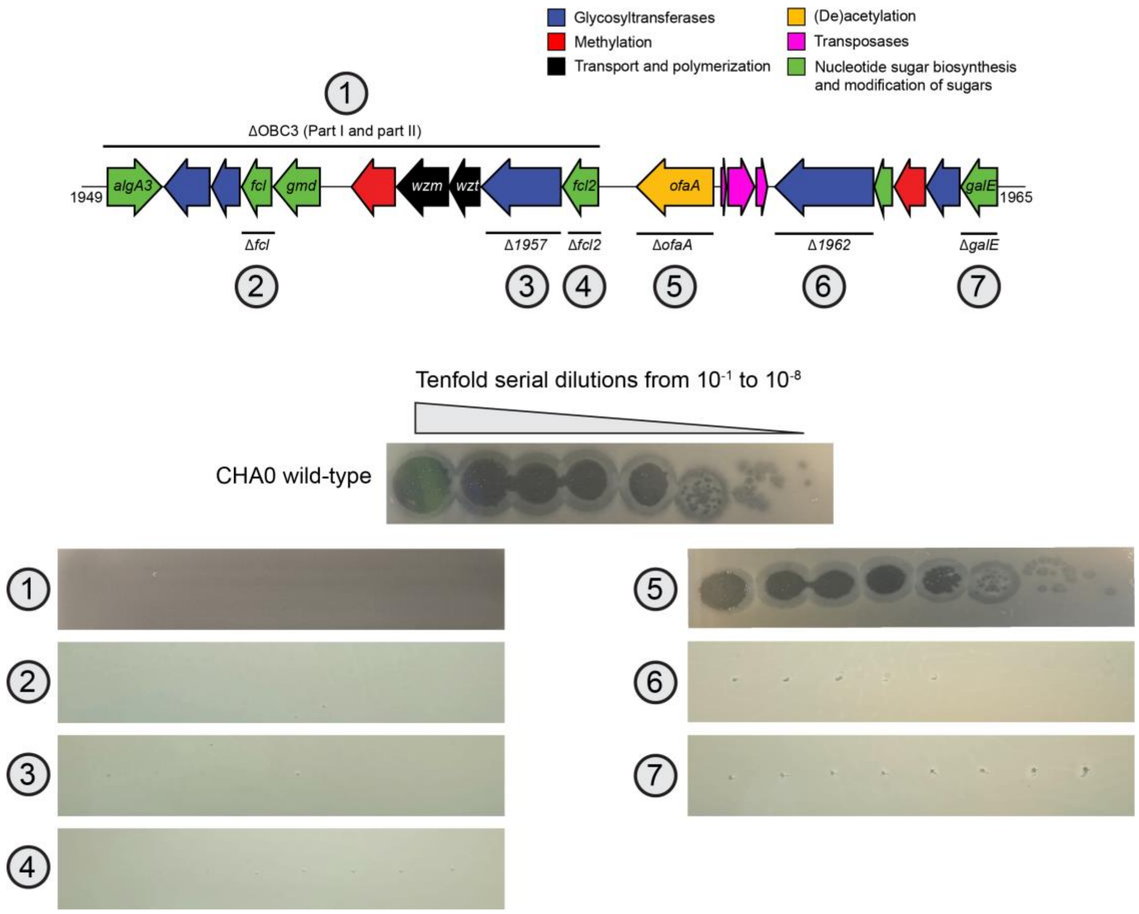

Fig. S5. Impact of multiple and individual gene deletions within the OBC3 gene cluster of *Pseudomonas protegens* CHA0 on its phage susceptibility. Spot assays of the phage ΦGP100 onto a bacterial lawn of CHA0 wild type or mutants. The deletion of the *ofaA* gene encoding an O-antigen acetylase has no influence on the sensitivity of CHA0 towards ΦGP100.

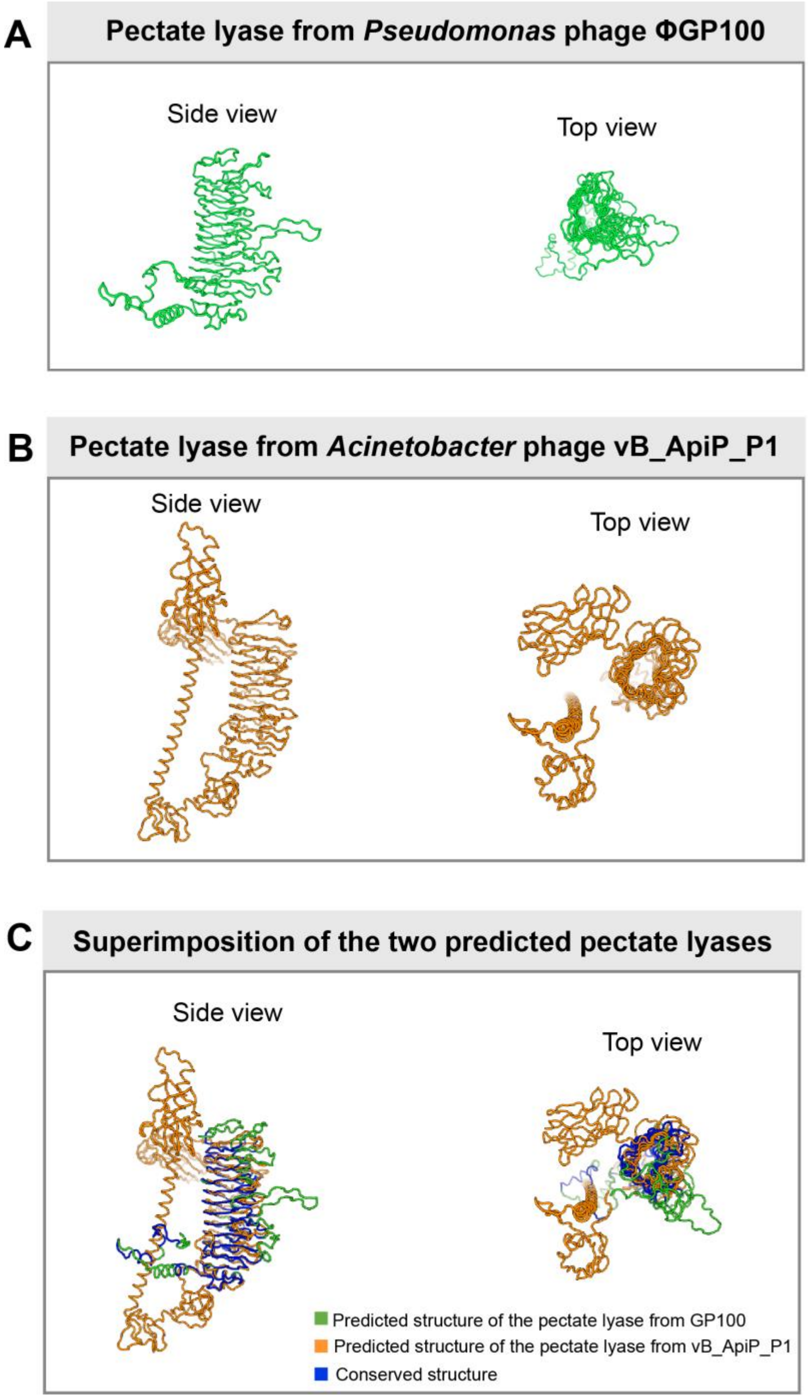

Fig. S6. Prediction and protein structure comparison of the putative pectate lyase encoded in the genome of the phage ΦGP100. (A) AlphaFold2 prediction of the protein structure of the pectate lyase of the phage ΦGP100.**(B)** AlphaFold2 prediction of the protein structure of the pectate lyase of the phage vB_ApiP_P1 previously demonstrated to display a depolymerase activity towards *Acinetobacter* cells (1). **(C)** Superimposition of the two pectate lyases shows a conserved structure.

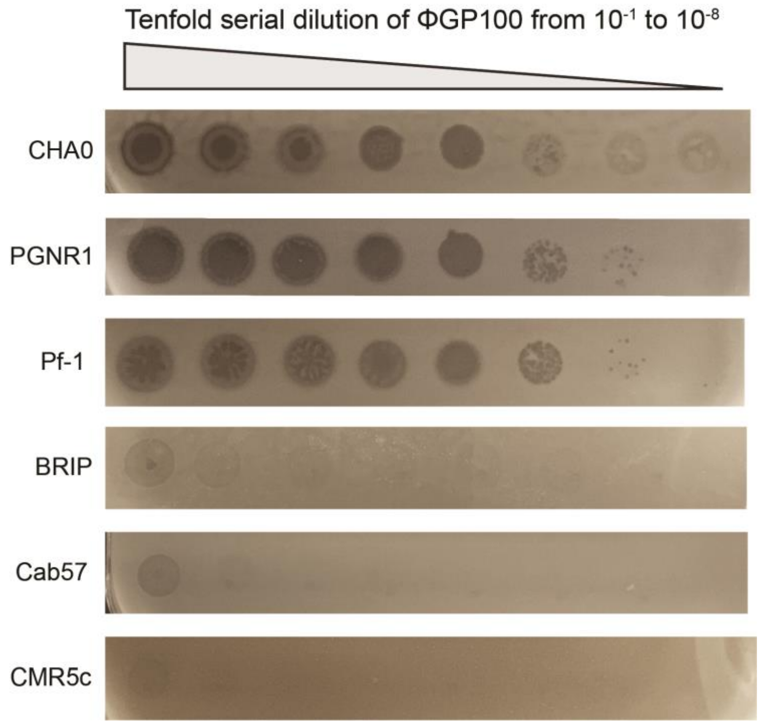

Fig. S7. Different sensitivity patterns displayed by *Pseudomonas protegens* subgroup strains that harbor the OBC3 gene cluster. Spot assays of ΦGP100 onto bacterial lawns of the different *Pseudomonas* strains. Strains CHA0, PGNR1 and Pf1 exhibit a similar sensitivity pattern towards the ΦGP100 phage, while strains BRIP, Cab57 and CMR5c display a moderate to mild sensitivity.

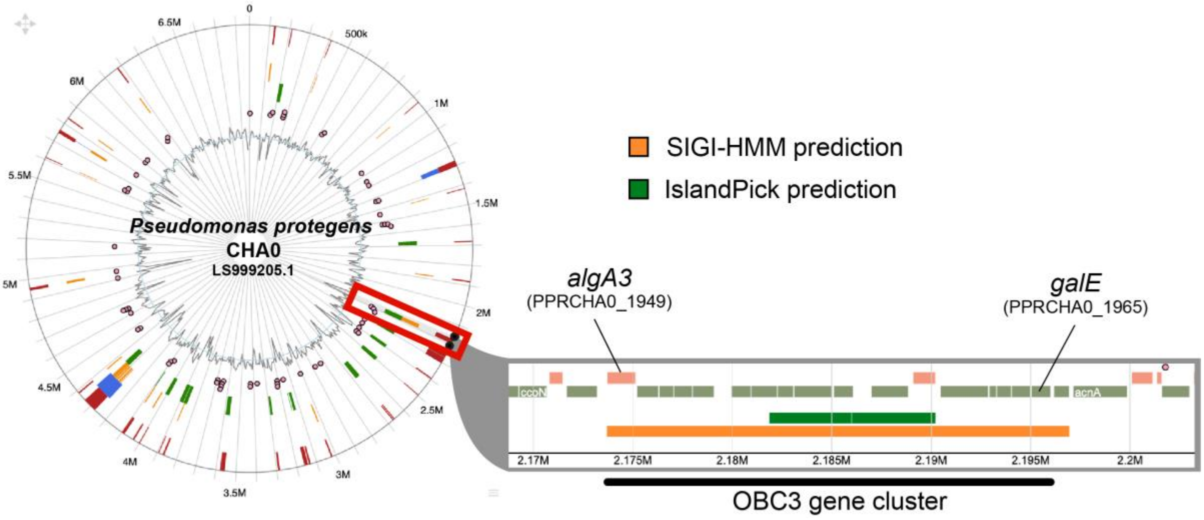

Fig. S8. The OBC3 gene cluster is located within a potential genomic island. Predictions were obtained from IslandViewer4 (https://www.pathogenomics.sfu.ca/islandviewer/). The location of the OBC3 gene cluster is highlighted with a red square.

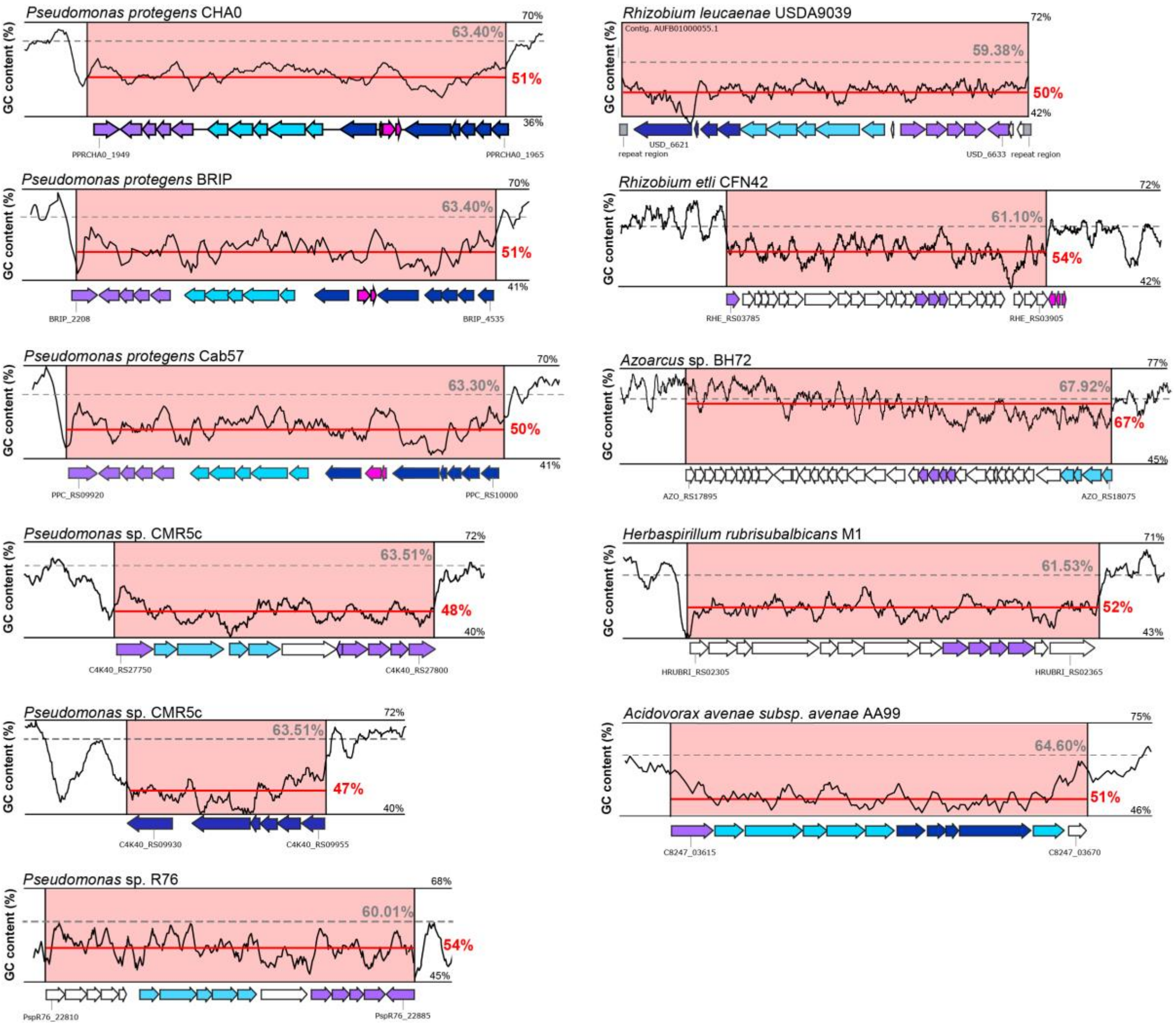

Fig. S9. Horizontal gene transfer signature detected within the different orthologous genomic regions of OBC3 in bacterial genomes. The gray dashed lines represent the average GC content of the whole genome, while the red lines correspond to the GC content of the orthologous genomic regions of the OBC3 gene cluster. Dark lines illustrate the GC skew calculated using a 500-bp window frame.

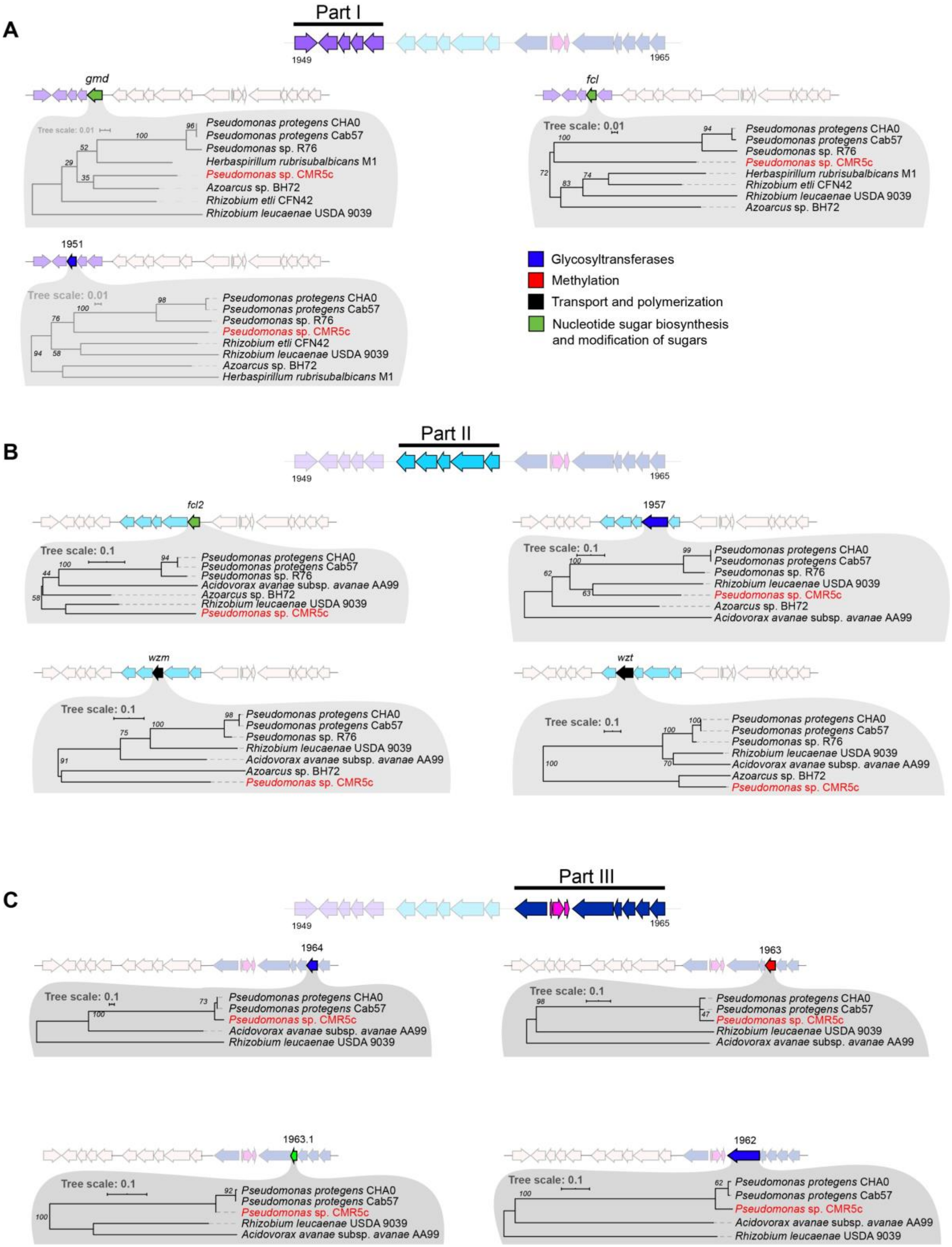

Fig. S10. Maximum likelihood trees of the amino acid sequences of the corresponding genes conserved within the OBC3 gene cluster detected in bacterial genomes. Maximum likelihood trees obtained from the aligned amino acid sequences present within the three parts of the OBC3 gene cluster (**A**: part 1; **B**: part 2; **C**: part 3). The robustness of each tree was assessed with 100 bootstraps replicates. *Pseudomonas* strains with names written in red exhibit incongruences in the tree topology compared to the species phylogenetic tree shown in **Fig. 4**.

Supplementary tables

Table S1. Plasmids used in this study.

Plasmid	Genotype or relevant characteristics[1]	Reference or source
pEMG	Expression vector; *ori*R6K, *lacZα* with two flanking I-SceI sites; Km^R, Ap^R	(2)
pRL27	Tn5-RL27 (Km^R-*ori*R6 K) delivery vector for the generation of transposon mutant library.	(3)
pME8300	Carrier plasmid for Tn*7* for $P_{tac/lacIq}$ controlled target gene expression; Gm^r, Ap^r	(4)
pUX-BF13	Helper plasmid encoding Tn*7* transposition functions; R6K-replicon; Ap^r	(4)
pME497	Mobilizing plasmid; IncP-1 Tra RepA (Ts); Ap^R	(5)
pME11021	pME8300-$P_{tac/lacIq}$-*fcl*(CFN42); IPTG-inducible expression of *Rhizobium etli* CFN42 *fcl* (RHE_CH00763); Gm^r, Ap^r	This study
pME11022	pME8300-$P_{tac/lacIq}$-*fcl*(CHA0); IPTG-inducible expression of *Pseudomonas protegens* CHA0 *fcl*; Gm^r, Ap^r	This study
pME11107	pEMG::*Δfcl2*; suicide plasmid for the deletion of the *fcl2*; Km^R	This study
pME11108	pEMG::*Δ1962*; suicide plasmid for the deletion of PPRCHA0_1962; Km^R	This study
pME11106	pEMG::*ΔgalE*; suicide plasmid for the deletion of the *galE* gene; Km^R	This study
pME11105	pEMG::*ΔoatA*; suicide plasmid for the deletion of the *oatA* gene; Km^R	This study
pME11123	pEMG::*algC;* suicide plasmid for the deletion of the *algC* gene; Km^R	This study

[1] Ap^R, ampicillin resistance; Cm^R, chloramphenicol resistance; Gm^R, gentamycin resistance; Km^R, kanamycin resistance; Tc^R, tetracycline resistance.

Table S2. Tn-seq characteristics.

Sample	Biological replicate	Total number of reads obtained	Number of HQ reads [a]	Total of mapping HQ reads [b]	Mean read length (bp)	Total insertion hits	Number of insertions every 1,000 bp [c]	Total genes with hits	Mean of hits per genes
Control	1	20,242,678	11,731,031	9,769,299 (83%)	113.3	526,158	77	5,788 (94%)	82
Control	2	22,399,901	13,419,248	11,237,426 (84%)	112.6	425,745	63	5,699 (93%)	67
Control	3	22,967,708	13,804,616	11,411,687 (83%)	113.2	546,931	77	5,854 (95%)	84
MOI of 1 [d]	1	26,759,802	11,170,004	9,282,169 (83%)	112.9	55,159	-	4,888 (79%)	10
MOI of 1	2	22,880,585	9,139,588	7,694,614 (84%)	113.6	75,459	-	5,681 (92%)	12
MOI of 1	3	23,263,048	13,171,418	11,238,630 (85%)	113.9	52,282	-	4,649 (76%)	10
MOI of 10	1	20,814,448	11,854,767	9,855,684 (83%)	112.9	46,562	-	4,605 (75%)	9
MOI of 10	2	20,297,964	10,598,935	8,989,953 (85%)	112.5	49,067	-	4,815 (78%)	9
MOI of 10	3	16,808,770	9,302,312	7,851,007 (84%)	113.5	42,104	-	4,425 (72%)	9
MOI of 100	1	20,665,244	9,817,868	8,286,692 (84%)	112.5	44,272	-	4,581 (74%)	9
MOI of 100	2	18,191,640	10,076,908	8,619,761 (86%)	114.1	44,946	-	4,570 (74%)	9
MOI of 100	3	21,690,076	12,089,051	10,315,697 (85%)	113.9	46,204	-	4,760 (77%)	9

[a] HQ reads, high-quality reads (i.e., trimmed reads and reads that matched with the Tn*5* transposon sequence).

[b] Number of high-quality reads that mapped to the genome of CHA0 (LS999205.1).

[c] Total number of genes in the genome of *P. protegens* CHA0: 6,158.

[d] MOI, multiplicity of infection.

Table S3. Phage genomes used in this study.

Accession Number	Taxid	Genome length (bp)	Name of the phage	Type	Phage family	Bacterial host group	Bacterial host name
NC_003907	2907796	46,012	*Vibrio* phage VpV262	dsDNA	*Zobellviridae*	Gammaproteobacteria	*Vibrio parahaemolyticus*
NC_048671	2847823	40,907	*Lentibacter* phage vB_LenP_ICBM2	dsDNA	*Zobellviridae*	Alphaproteobacteria	*Lentibacter sp.*
NC_018280	1197951	38,889	*Celeribacter* phage P12053L	dsDNA	*Zobellviridae*	Alphaproteobacteria	*Celeribacter marinus*
NC_002519	2905867	39,898	*Roseobacter* phage SIO1	dsDNA	*Zobellviridae*	Alphaproteobacteria	*Roseobacter*
NC_048672	2847822	40,163	*Lentibacter* phage vB_LenP_ICBM1	dsDNA	*Zobellviridae*	Alphaproteobacteria	*Lentibacter sp.*
NC_024791	1529058	50,440	*Vibrio* phage ICP2_2013_A_Haiti	dsDNA	*Zobellviridae*	Gammaproteobacteria	*Vibrio cholerae*
NC_015158	979533	49,675	*Vibrio* phage ICP2	dsDNA	*Zobellviridae*	Gammaproteobacteria	*Vibrio cholerae*
KM224878	1529057	50,250	*Vibrio* phage ICP2_2011_A	dsDNA	*Zobellviridae*	Gammaproteobacteria	*Vibrio cholerae*
HQ641346	979534	48,626	*Vibrio* phage ICP2_2006_A	dsDNA	*Zobellviridae*	Gammaproteobacteria	*Vibrio cholerae*
NC_021300	1316739	43,882	*Pseudoalteromonas* phage RIO-1	dsDNA	*Zobellviridae*	Gammaproteobacteria	*Pseudoalteromonas marina*
NC_048630	1357706	45,035	*Pseudoalteromonas* phage HP1	dsDNA	*Zobellviridae*	Gammaproteobacteria	*Pseudoalteromonas sp.*
NC_019540	1161935	49,390	*Salinivibrio* phage CW02	dsDNA	*Zobellviridae*	Gammaproteobacteria	*Salinivibrio costicola*
NC_027988	1622234	47,636	*Citrobacter* phage CVT22	dsDNA	*Zobellviridae*	Gammaproteobacteria	*Citrobacter sp.* TM1552
LT986460	2055238	50,547	*Pseudomonas* phage GP100	dsDNA	*Zobellviridae*	Gammaproteobacteria	*Pseudomonas protegens*
NC_007808	347327	49,639	*Pseudomonas* phage PA11	dsDNA	*Zobellviridae*	Gammaproteobacteria	*Pseudomonas aeruginosa*
NC_031274	1784982	50,509	*Pseudomonas* phage O4	dsDNA	*Zobellviridae*	Gammaproteobacteria	*Pseudomonas aeruginosa* PAO1
NC_018859	1604355	77,315	*Escherichia* phage ECBP2	dsDNA	-	Gammaproteobacteria	*Escherichia coli*
NC_023593	1436889	76,184	*Escherichia* phage KBNP1711	dsDNA	-	Gammaproteobacteria	*Escherichia coli*
NC_027395	1519788	77,327	*Escherichia* phage vB_EcoP_SU10	dsDNA	-	Gammaproteobacteria	*Escherichia coli*
NC_010324	2679905	77,554	*Escherichia* phage phiEco32	dsDNA	-	Gammaproteobacteria	*Escherichia coli*
NC_018835	1237159	77,448	*Escherichia* phage NJ01	dsDNA	-	Gammaproteobacteria	*Escherichia coli*
NC_028903	1598146	77,266	*Escherichia* phage 172-1	dsDNA	-	Gammaproteobacteria	*Escherichia coli*
NC_015938	1054968	89,916	*Salmonella* phage 7-11	dsDNA	*Podoviridae*	Gammaproteobacteria	*Salmonella enterica subsp. enterica serovar Newport*
NC_019402	1141137	76,631	*Cronobacter* phage vB_CsaP_GAP52	dsDNA	*Podoviridae*	Gammaproteobacteria	*Cronobacter sakazakii*
NC_048861	2712958	90,710	*Proteus* phage Privateer	dsDNA	-	Gammaproteobacteria	*Proteus mirabilis*
NC_048664	2699738	92,122	*Cronobacter* phage vB_CsaP_009	dsDNA	-	Gammaproteobacteria	*Cronobacter sakazakii*

NC_048776	2591033	97,988	*Aeromonas* phage LAh_9	dsDNA	-	Gammaproteobacteria	*Aeromonas hydrophila*
NC_048773	2588517	102,915	*Aeromonas* phage 4_4572	dsDNA	-	Gammaproteobacteria	*Aeromonas hydrophila subsp. hydrophila*
NC_048775	2591032	97,408	*Aeromonas* phage LAh_8	dsDNA	-	Gammaproteobacteria	*Aeromonas hydrophila*
NC_048774	2591030	101,390	*Aeromonas* phage LAh_6	dsDNA	-	Gammaproteobacteria	*Aeromonas hydrophila*
KJ936628	1524880	50,431	*Vibrio* phage VPp1	dsDNA	*Podoviridae*	Gammaproteobacteria	*Vibrio parahaemolyticus*
NC_042103	2079288	45,936	*Pseudomonas* phage Bjorn	dsDNA	-	Gammaproteobacteria	*Pseudomonas* sp.
NC_017971	1114179	46,271	*Pseudomonas* phage tf	dsDNA	-	Gammaproteobacteria	*Pseudomonas putida*
NC_018850	1235661	45,517	*Pseudomonas* phage UFV-P2	dsDNA	-	Gammaproteobacteria	*Pseudomonas fluorescens*
NC_042107	2079543	45,058	*Pseudomonas* phage NV1	dsDNA	-	Gammaproteobacteria	*Pseudomonas tolaasii* NCPPB 2192
NC_028933	1589273	45,626	*Pseudomonas* phage PhiCHU	dsDNA	-	Gammaproteobacteria	*Pseudomonas aeruginosa*
NC_004466	2905964	45,503	*Pseudomonas* phage PaP3	dsDNA	-	Gammaproteobacteria	*Pseudomonas*
JN254801	1158721	44,789	*Pseudomonas* phage MR299-2	dsDNA	-	Gammaproteobacteria	*Pseudomonas aeruginosa*
NC_019813	1234701	44,030	*Pseudomonas* phage vB_PaeP_p2-10_Or1	dsDNA	-	Gammaproteobacteria	*Pseudomonas aeruginosa*
HE983844	1229675	45,469	*Pseudomonas* phage vB_PaeP_C1-14_Or	dsDNA	-	Gammaproteobacteria	*Pseudomonas aeruginosa*
NC_026599	1548906	45,808	*Pseudomonas* phage vB_PaeP_C2-10_Ab22	dsDNA	-	Gammaproteobacteria	*Pseudomonas aeruginosa*
NC_023583	1406974	45,696	*Pseudomonas* phage TL	dsDNA	-	Gammaproteobacteria	*Pseudomonas aeruginosa*
NC_010325	484895	45,625	*Bruynoghevirus* LUZ24	dsDNA	-	Gammaproteobacteria	*Pseudomonas aeruginosa*
NC_042343	1273709	43,895	*Pseudomonas* phage PaP4	dsDNA	-	Gammaproteobacteria	*Pseudomonas aeruginosa*
NC_022971	1429758	45,344	*Pseudomonas* phage phiIBB-PAA2	dsDNA	-	Gammaproteobacteria	*Pseudomonas aeruginosa*
NC_028919	1640969	45,673	*Pseudomonas* phage DL54	dsDNA	-	Gammaproteobacteria	*Pseudomonas aeruginosa* PAO1

Table S4. Update of the phage ΦGP100 annotation.

Locus_tag	Start	End	Size (bp)	Strand	Predicted function [Associated Source Organism]	Accession	Identity %
GP100_00001	424	573	149	+	Hypothetical protein		
GP100_00002	573	644	71	+	Hammerhead_II		
GP100_00003	1570	1833	263	+	Hypothetical protein		
GP100_00004	2034	2106	72	+	tRNA-Stop(tta)		
GP100_00005	2129	2689	560	+	Coil containing protein [*Vibrio* phage 1.204.O._10N.222.46.F12]	YP_009817597.1	43/110 (39%)
GP100_00006	2820	2966	146	+	Hypothetical protein		
GP100_00007	2966	3163	197	+	Hypothetical protein		
GP100_00008	3391	3729	338	+	Hypothetical protein		
GP100_00009	3779	3940	161	+	Hypothetical protein		
GP100_00010	4265	4480	215	+	Hypothetical protein		
GP100_00011	4489	4779	290	+	Hypothetical protein		
GP100_00012	4926	5072	146	+	Hypothetical protein		
GP100_00013	5072	5392	320	+	Hypothetical protein		
GP100_00014	5593	5928	335	+	Hypothetical protein		
GP100_00015	5978	6175	197	+	Hypothetical protein		
GP100_00016	6148	6288	140	-	Hypothetical protein		
GP100_00017	6315	6449	134	+	Hypothetical protein		
GP100_00018	6449	6634	185	+	Hypothetical protein		
GP100_00019	6727	6930	203	+	Hypothetical protein		
GP100_00020	6923	7876	953	+	Hypothetical protein		
GP100_00021	7869	8504	635	+	Putative amidotransferase [*Pseudomonas* phage O4]	YP_009304537.1	116/213 (54%)
GP100_00022	8501	8719	218	+	Hypothetical protein		
GP100_00023	8774	10147	1373	+	Putative amidotransferase [*Pseudomonas* phage O4]	YP_009304535.1	158/415 (38%)
GP100_00024	10180	10647	467	+	Putative HNH endonuclease [*Escherichia* phage LL11]	YP_009812380.1	60/148 (41%)
GP100_00025	10637	11506	869	+	Putative COOH-NH2 ligase [*Pseudomonas* phage O4]	YP_009304534.1	168/275 (61%)

GP100_00026	11508	12065	557	+	Hypothetical protein		
GP100_00027	12062	12946	884	+	Putative amidoligase [*Pseudomonas* phage O4]	YP_009304532.1	169/275 (61%)
GP100_00028	12946	13155	209	+	Hypothetical protein		
GP100_00029	13152	14285	1133	+	Putative ATP-grasp protein [*Pseudomonas* phage O4]	YP_009304531.1	141/301 (47%)
GP100_00030	14353	14565	212	+	Hypothetical protein		
GP100_00031	14574	14750	176	+	Hypothetical protein		
GP100_00032	14762	16483	1721	+	Putative helicase/primase [*Pseudomonas* phage O4]	YP_009304525.1	398/570 (70%)
GP100_00033	16483	16650	167	+	Recombination protein RecR		
GP100_00034	16637	17125	488	+	RNA polymerase sigma factor [*Pseudomonas* phage O4]	YP_009304524.1	73/159 (46%)
GP100_00035	17115	18992	1877	+	Putative DNA polymerase [*Pseudomonas* phage O4]	YP_009304521.1	389/625 (62%)
GP100_00036	18976	19077	101	+	Hypothetical protein		
GP100_00037	19077	19430	353	+	Hypothetical protein		
GP100_00038	19434	20141	707	+	Putative ssDNA binding protein [*Pseudoalteromonas* phage RIO-1]	YP_008051092.1	65/193 (34%)
GP100_00039	20135	20320	185	+	Hypothetical protein		
GP100_00040	20392	20766	374	+	Hypothetical protein		
GP100_00041	20757	20942	185	+	Hypothetical protein		
GP100_00042	20926	21804	878	+	Putative 5'-3' exonuclease [*Pseudomonas* phage O4]	YP_009304516.1	166/286 (58%)
GP100_00043	21797	22336	539	+	Metallophosphoesterase [*Ralstonia* phage RSP15]	YP_009277057.1	82/181 (45%)
GP100_00044	22336	22869	533	+	Hypothetical protein		
GP100_00045	23040	23192	152	+	Hypothetical protein		
GP100_00046	23176	23619	443	+	Putative terminase small subunit [*Pseudomonas* phage O4]	YP_009304514.1	89/134 (66%)
GP100_00047	23606	23860	254	+	Hypothetical protein		
GP100_00048	23857	24192	335	+	Putative AAT_I superfamily protein [*Pectobacterium* phage PPWS2]	YP_009816196.1	34/88 (39%)
GP100_00049	24179	24952	773	+	Putative 3'-5' exonuclease [*Pseudomonas* phage O4]	YP_009304511.1	193/252 (77%)
GP100_00050	25166	25534	368	+	Putative holin [*Pseudomonas* phage UFV-P2]	YP_007518479.1	64/87 (74%)
GP100_00051	25620	26225	605	+	PhoH-like protein [*Pseudomonas* phage PA11]	YP_001294617.1	155/195 (79%)
GP100_00052	26225	26527	302	+	Hypothetical protein		
GP100_00053	26521	26670	149	+	Hypothetical protein		
GP100_00054	26667	26861	194	+	Hypothetical protein		

GP100_00055	26900	27163	263	+	Neck appendage protein precursor [*Bacillus* phage GA1]	NP_073695	17/65 (26%)
GP100_00056	27199	27483	284	-	Hypothetical protein		
GP100_00057	27576	28049	473	-	Hypothetical protein		
GP100_00058	28027	28266	239	-	Hypothetical protein		
GP100_00059	28269	28703	434	-	Putative endolysin [*Pseudomonas* phage O4]	YP_009304499.1	118/143 (83%)
GP100_00060	28703	28807	104	-	Hypothetical protein		
GP100_00061	28809	30641	1832	-	Putative IVP-C-like protein [*Klebsiella* phage vB_KpnP_Klyazma]	UVD32011.1	177/613 (29%)
GP100_00062	30643	35430	4787	-	Putative IVP-D-like protein [*Klebsiella* phage vB_KpnP_Klyazma]	UVD32010.1	520/1652 (31%)
GP100_00063	35443	35964	521	-	Hypothetical protein		
GP100_00064	35969	36415	446	-	Acyl-CoA N-acyltransferase (GNAT) domain protein [*Lentibacter* virus vB_LenP_ICBM1]	YP_009834282.1	26/84 (31%)
GP100_00065	36408	38705	2297	-	Putative head closure protein [*Pseudomonas* phage O4]	YP_009304494.1	407/772 (53%)
GP100_00066	38689	38871	182	-	Hypothetical protein		
GP100_00067	38871	39677	806	-	Putative adaptor protein [*Pseudomonas* phage O4]	YP_009304492.1	152/259 (59%)
GP100_00068	39677	40303	626	-	Neck protein [*Raoultella* phage RP180]	YP_009821849.1	58/151 (38%)
GP100_00069	40408	41052	644	-	Putative tail shaft protein Tsh [*Listeria* phage vB_LmoS_188]	YP_009204855.1	40/94 (43%)
GP100_00070	41068	42021	953	-	Putative capsid protein [*Pseudomonas* phage O4]	YP_009304488.1	234/311 (75%)
GP100_00071	42034	42792	758	-	Putative scaffold protein [*Pseudomonas* phage O4]	YP_009304487.1	119/252 (47%)
GP100_00072	42902	42976	74	-	tRNA-Asn(gtt)		
GP100_00073	43104	43343	239	-	Hypothetical protein		
GP100_00074	43346	45100	1754	-	Putative portal protein [*Pseudomonas* phage O4]	YP_009304485.1	422/584 (72%)
GP100_00075	45110	46714	1604	-	Putative terminase large subunit [*Pseudomonas* phage O4]	YP_009304484.1	397/533 (74%)
GP100_00076	46714	47214	500	-	Hypothetical protein		
GP100_00077	47250	48761	1511	-	Putative pectate lyase (potential depolymerase)		
GP100_00078	48761	49684	923	-	Hypothetical protein		
GP100_00079	49681	50328	647	-	Hypothetical protein		

Table S5. Bacterial strains used in this study.

Strain [a]	Genome accession no.	Origin	Reference or source
***Pseudomonas protegens* subgroup**			
P. protegens BRIP	NZ_LHUW00000000.1	Cyclops	(6, 7)
P. protegens Cab57	NZ_AP014522.1	Shepperd's purse rhizosphere	(8)
P. protegens CHA0^{T}	LS999205.1	Tobacco rhizosphere	(9, 10)
P. protegens K94.41	NZ_LHUU00000000.1	Cucumber rhizosphere	(7, 11)
P. protegens Pf-5	NC_004129.6	Cotton rhizosphere	(12, 13)
P. protegens PF	NZ_LHUX00000000.1	Wheat leaves	(7, 14)
P. protegens Pf1	ERS3935804	Tobacco	(14)
P. protegens PGNR1	NZ_LHUV00000000.1	Tobacco rhizosphere	(7, 14)
P. saponiphila DMS9751^{T}	FNTJ00000000.1	unknown	(15)
Pseudomonas sp. AU11706	NZ_LCZB00000000.1	Cystic fibrosis sputum	(16)
Pseudomonas sp. AU20219	NZ_LDET00000000.1	Cystic fibrosis sputum	(16)
Pseudomonas sp. AU13852	NZ_LCZC00000000.1	Cystic fibrosis sputum	(16)
Pseudomonas sp. CMR5c	NZ_LHUY00000000.1	Red cocoyam rhizosphere	(7, 17)
Pseudomonas sp. CMR12a	CP027706.1	Red cocoyam rhizosphere	(7, 17)
Pseudomonas sp. Pf4	LUUD00000000.1	Lamb's lettuce	(18)
Pseudomonas sp. Pf11	LUUE00000000.1	Lamb's lettuce	(18)
Pseudomonas sp. LD120	ERR3588830	Blade of marine alga	(19, 20)
Pseudomonas sp. Os17	NZ_AP014627.1	Rice rhizosphere	(8)
Pseudomonas sp. St29	NZ_AP014628.1	Potato rhizosphere	(8)
***Pseudomonas chlororaphis* subgroup**			
P. chlororaphis O6	NZ_CM001490.1	Soil	(21)
P. chlororaphis R47	Genome not published	Potato rhizosphere	(22)
P. chlororaphis subsp. *aureofaciens* 30-84	NZ_CM001559.1	Wheat rhizosphere	(21, 23)
P. chlororaphis subsp. *aurantiaca* JD37	NZ_CP009290.1	Potato rhizosphere	(24, 25)
P. chlororaphis subsp. *aureofaciens* CD	NZ_LHVB00000000.1	Cyclops (water)	(6, 7)
P. chlororaphis subsp. *aureofaciens* LMG1245^{T}	NZ_LHVA00000000.1	River clay	(7, 26)
P. chlororaphis subsp. *chlororaphis* LMG5004^{T}	NZ_LHVC00000000.1	Contaminated plate	(7, 27)
P. chlororaphis subsp. *piscium* DSM21509^{T}	NZ_CP027707.1	European perch intestine	(28)
P. chlororaphis subsp. *piscium* PCL1391	NZ_CP027736.1	Tomato root	(7, 29)
**Other species**			
Acidovorax avenae subsp. *avenae* AA99	CP028289.1	Maize	(30)
Azoarcus sp. BH72	NC_008702.1	Kallar grass	(31)
Herbaspirillum rubrisubalbicans M1	CP013737.1	Sugar cane	(32)
Rhizobium etli CFN42^{T}	CP000133.1	Common bean	(33)
Rhizobium leucaenae USDA 9039^{T}	AUFB01000000.1	*Leucaena esculenta*	(34)

[a] ^{T}, type strain

Table S6. Occurrence of inverted repeats sequences inside the OBC3 gene cluster of *Pseudomonas protegens* CHA0.

Inverted repeat	Stem	Loop	Sequence	Occurrence inside the genome of CHA0
IR1	17	9,928	5'-CGCTTTTCAAACTCTAC-3'<br>3'-GCGAAAAGTTTGAGATG-5'	Unique
IR2	13	15,271	5'-AGCTGAAGGCAGT-3'<br>3'-TCGACTTCCGTCA-5'	Unique
IR3	13	7,086	5'-GCAGGCGACGCAA-3'<br>3'-CGTCCGCTGCGTT-5'	Unique
IR4	13	4,123	5'-TGCGAAAGACTAC-3'<br>3'-ACGCTTTCTGATG-5'	Unique
IR5	13	7,221	5'-TTGCATCGCTTGG-3'<br>3'-AACGTAGCGAACC-5'	Unique
IR6	12	3,406	5'-ATTAGCGGCGCA-3'<br>3'-TAATCGCCGCGT-5'	Unique
IR7	12	13,520	5'-CTAACTATTACT-3'<br>3'-GATTGATAATGA-5'	Unique
IR8	12	2,975	5'-GCTTTGCTACTG-3'<br>3'-CGAAACGATGAC-5'	Unique
IR9	12	8,540	5'-TTTGGTCGCTTC-3'<br>3'-AAACCAGCGAAG-5'	Unique
IR10	12	15,176	5'-TTTTGGCAGCCC-3'<br>3'-AAAACCGTCGGG-5'	Three times
IR11	12	8,568	5'-ACGCCCTGAAAA-3'<br>3'-TGCGGGACTTTT-5'	Four times
IR12	12	4	5'-ATCCAGCGGCGG-3'<br>3'-TAGGTCGCCGCC-5'	Six times
IR13	12	5,410	5'-TGAAGCTGGCAG-3'<br>3'-ACTTCGACCGTC-5'	Six times
IR14	12	11,731	5'-TTCATTGACCAG-3'<br>3'-AAGTAACTGGTC-5'	Seven times

Table S7. *Pseudomonas protegens* CHA0 derivatives and *Escherichia coli* strain used in this study.

**Strain name**	**Strain code**	**Genotype or relevant characteristics**[a]	**Reference or source**
Pseudomonas protegens **CHA0 and derivatives**			
CHA0^T	CHA0^T	*P. protegens* type strain; wild type; genome accession no. LS999205.1	(9, 10)
Δ*fcl*	CHA5205	CHA0 with deletion of PPRCHA0_1952	(35)
Δ*wbpL*	CHA5161	CHA0 with deletion of PPRCHA0_4350	(35)
Δ*wzx*	CHA5206	CHA0 with deletion of PPRCHA0_4354	(35)
Δ*fcl2*	CHA5328	CHA0 with deletion of PPRCHA0_1958	This study
Δ*galE*	CHA5327	CHA0 with deletion of PPRCHA0_1965	This study
Δ*1962*	CHA5330	CHA0 with deletion of PPRCHA0_1962	This study
Δobc3	CHA5182	CHA0 with deletion of PPRCHA0_1950 through PPRCHA0_1958	(35)
Δ*oatA*	CHA5329	CHA0 with deletion of PPRCHA0_1960	This study
Δ*algC*	CHA5303	CHA0 with deletion of PPRCHA0_5962	This study
CHA5211	CHA5211	CHA5205::attTn*7*-$P_{tac/lacIq}$-*fcl*(CFN42); Gm^r	This study
CHA5212	CHA5212	CHA5205::attTn*7*-$P_{tac/lacIq}$-*fcl*(CHA0); Gm^r	This study
Escherichia coli			
E. coli S17-1/λpir		Laboratory strain	(36)

[a] Gm^R, gentamicin resistance.

Table S8. Primers used in this study.

Name	Sequence 5' → 3', restriction enzyme(s)[a]	Purpose
del-fcl2-1	CGGAATTCTACCATTCGGGCCACATG, EcoRI	Deletion of *fcl2* (PPRCHA0_1958)
del-fcl2-2	GCTCTAGAATCTGATGCAAGCACCGC, XbaI	Deletion of *fcl2* (PPRCHA0_1958)
del-fcl2-3	GCTCTAGACATTCCTTTGTGCCCTGT, XbaI	Deletion of *fcl2* (PPRCHA0_1958)
del-fcl2-4	ACGCGTCGACATGTAACGCAGCACCTCT, SalI	Deletion of *fcl2* (PPRCHA0_1958)
fcl2-check-F	GAAGTGTGCGGGTAAGTG	Control of deletion of *fcl2* (PPRCHA0_1958)
fcl2-check-R	AATGTGCCAATGCCCTTG	Control of deletion of *fcl2* (PPRCHA0_1958)
del-algC-1	ggGGTACCATGCACGCTTTACAAGCC, KpnI	Deletion of *algC* (PPRCHA0_5962)
del-algC-2	cgGGATCCCATCACTGAGGGTGCTCC, BamHI	Deletion of *algC* (PPRCHA0_5962)
del-algC-3	gGGATCCTGATTGTTTGAAGCACCG, BamHI	Deletion of *algC* (PPRCHA0_5962)
del-algC-4	acgcGTCGACGATGTTGTAGGACTCGCC, SalI	Deletion of *algC* (PPRCHA0_5962)
algC-check-F	GCCAGCTGATCTCTTTTATC	Control of deletion of *algC* (PPRCHA0_5962)
algC-check-R	TTTTCGGCTTTCAATGCTTC	Control of deletion of *algC* (PPRCHA0_5962)
del-oatA-1	CGGAATTCCGATGTTACCACGTCGTA, EcoRI	Deletion of *oatA* (PPRCHA0_1959)
del-oatA-2	CGAGCTCTAACGCCTCAATACGTTATCA, SacI	Deletion of *oatA* (PPRCHA0_1959)
del-oatA-3	CGAGCTCCATGTCTTGGAAAAATCCTTG, SacI	Deletion of *oatA* (PPRCHA0_1959)
del-oatA-4	GGGGTACCTCCTTGGCAAAGTATGCG, KpnI	Deletion of *oatA* (PPRCHA0_1959)
oatA-check-F	TTCCTGTAGCCCTTCTGC	Control of deletion of *oatA* (PPRCHA0_1959)
oatA-check-R	GCGCGCAGCTTGATAAAG	Control of deletion of *oatA* (PPRCHA0_1959)
del-1962-1	CGGAATTCAATTTGATTGCCAGCATGAGT, EcoRI	Deletion of PPRCHA0_1962
del-1962-2	GGGGTACCCTTTGATGGCGAGCGTTG, KpnI	Deletion of PPRCHA0_1962
del-1962-3	GGGGTACCCATCCTGGAATGTACCTTGTG, KpnI	Deletion of PPRCHA0_1962
del-1962-4	ACGCGTCGACATGATTGCACTGGCCATT, SalI	Deletion of PPRCHA0_1962
1962-check-F	CAGTACAGCAGCTCCGAT	Control of deletion of PPRCHA0_1962
1962-check-R	CGCACTAGGTGCTCAGTA	Control of deletion of PPRCHA0_1962
del-galE-1	CGGAATTCATCTGCAGGGATGTCGAT, EcoRI	Deletion of *galE* (PPRCHA0_1965)
del-galE-2	GGGGTACCTAACGCAAATGACGCAATCTT, KpnI	Deletion of *galE* (PPRCHA0_1965)
del-galE-3	GGGGTACCCACACCGCCTCTCCTTCG, KpnI	Deletion of *galE* (PPRCHA0_1965)
del-galE-4	ACGCGTCGACTTGACCCTGACGGCAATT, SalI	Deletion of *galE* (PPRCHA0_1965)
galE-check-F	CTCGACGACTCATCAGGC	Control of deletion of *galE* (PPRCHA0_1965)
galE-check-R	TATGCCCGTGGATTGTTG	Control of deletion of *galE* (PPRCHA0_1965)
8300-F	ACATCATAACGGTTCTGGCAAA	Amplification of pME8300 to check insertions
8300-R	AAAAAGGGGACCTCTAGGGTC	Amplification of pME8300 to check insertions
pEMG check-F	GTAAAACGACGGCCAGT	Sequencing verification of the pEMG-based plasmids
pEMG check-R	AACAGCTATGACCATG	Sequencing verification of the pEMG-based plasmids

[a] Restriction sites are underlined.

References

1. Ojeda KJ, Simonds L, Noel KD. 2013. Roles of predicted glycosyltransferases in the biosynthesis of the *Rhizobium etli* CE3 O antigen. J Bacteriol 195:1949–1958.

2. Martínez-García E, de Lorenzo V. 2011. Engineering multiple genomic deletions in Gram-negative bacteria: analysis of the multi-resistant antibiotic profile of *Pseudomonas putida* KT2440. Environ Microbiol 13:2702–2716.

3. Larsen RA, Wilson MM, Guss AM, Metcalf WW. 2002. Genetic analysis of pigment biosynthesis in X*anthobacter autotrophicus* Py2 using a new, highly efficient transposon mutagenesis system that is functional in a wide variety of bacteria. Arch Microbiol 178:193–201.

4. Kupferschmied P, Péchy-Tarr M, Imperiali N, Maurhofer M, Keel C. 2014. Domain shuffling in a sensor protein contributed to the evolution of insect pathogenicity in plant-beneficial *Pseudomonas protegens*. PLoS Pathog 10:e1003964.

5. Schnider-Keel U, Seematter A, Maurhofer M, Blumer C, Duffy B, Gigot-Bonnefoy C, Reimmann C, Notz R, Défago G, Haas D, Keel C. 2000. Autoinduction of 2,4-diacetylphloroglucinol biosynthesis in the biocontrol agent *Pseudomonas fluorescens* CHA0 and repression by the bacterial metabolites salicylate and pyoluteorin. J Bacteriol 182:1215–1225.

6. Ruffner B, Péchy-Tarr M, Höfte M, Bloemberg G, Grunder J, Keel C, Maurhofer M. 2015. Evolutionary patchwork of an insecticidal toxin shared between plant-associated pseudomonads and the insect pathogens *Photorhabdus* and *Xenorhabdus*. BMC Genomics 16:609.

7. Flury P, Aellen N, Ruffner B, Péchy-Tarr M, Fataar S, Metla Z, Dominguez-Ferreras A, Bloemberg G, Frey J, Goesmann A, Raaijmakers JM, Duffy B, Höfte M, Blom J, Smits THM, Keel C, Maurhofer M. 2016. Insect pathogenicity in plant-beneficial pseudomonads: phylogenetic distribution and comparative genomics. ISME J 10:2527–2542.

8. Takeuchi K, Noda N, Katayose Y, Mukai Y, Numa H, Yamada K, Someya N. 2014. Rhizoxin analogs contribute to the biocontrol activity of newly isolated Pseudomonas strain. Mol Plant Microbe Interact. 28:333–342.

9. Stutz EW, Défago G, Kern H. 1986. Naturally occurring fluorescent pseudomonads involved in suppression of black root rot of tobacco. Phytopathol 76:181–185.

10. Smits THM, Rezzonico F, Frasson D, Vesga P, Vacheron J, Blom J, Pothier JF, Keel C, Maurhofer M, Sievers M. 2019. Updated genome sequence and annotation for the full genome of *Pseudomonas protegens* CHA0. Microbiol Res Announc 8:e01002-19.

11. Wang C, Ramette A, Punjasamarnwong P, Zala M, Natsch A, Moënne-Loccoz Y, Défago G. 2001. Cosmopolitan distribution of *phlD*-containing dicotyledonous crop-associated biocontrol pseudomonads of worldwide origin. FEMS Microbiol Ecol 37:105–116.

12. Howell CR, Stipanovic RD. 1979. Control of *Rhizoctonia solani* on cotton seedlings with *Pseudomonas fluorescens* and with an antibiotic produced by the bacterium. Phytopathol 69:480–482.

13. Paulsen IT, Press CM, Ravel J, Kobayashi DY, Myers GS, Mavrodi DV, DeBoy RT, Seshadri R, Ren Q, Madupu R. 2005. Complete genome sequence of the plant commensal *Pseudomonas fluorescens* Pf-5. Nat Biotechnol 23:873–878.

14. Keel C, Weller DM, Natsch A, Défago G, Cook RJ, Thomashow LS. 1996. Conservation of the 2,4-diacetylphloroglucinol biosynthesis locus among fluorescent *Pseudomonas* strains from diverse geographic locations. Appl Environ Microbiol 62:552–563.

15. Lang E, Burghartz M, Spring S, Swiderski J, Spröer C. 2010. *Pseudomonas benzenivorans* sp. nov. and *Pseudomonas saponiphila* sp. nov., represented by xenobiotics degrading type strains. Curr Microbiol 60:85–91.

16. Scales BS, Erb-Downward JR, LiPuma JJ, Huffnagle GB. 2015. Draft genome sequences of five *Pseudomonas fluorescens* subclade I and II strains, isolated from human respiratory samples. Genome Announc 3:e00837-15.

17. Perneel M, Heyrman J, Adiobo A, De Maeyer K, Raaijmakers JM, De Vos P, Höfte M. 2007. Characterization of CMR5c and CMR12a, novel fluorescent *Pseudomonas* strains from the cocoyam rhizosphere with biocontrol activity. J Appl Microbiol 103:1007–1020.

18. Polano C, Martini M, Savian F, Moruzzi S, Ermacora P, Firrao G. 2019. Genome sequence and antifungal activity of two niche-sharing *Pseudomonas protegens* related strains isolated from hydroponics. Microb Ecol 77:1025–1035.

19. Nagel K, Schneemann I, Kajahn I, Labes A, Wiese J, Imhoff JF. 2012. Beneficial effects of 2,4-diacetylphloroglucinol-producing pseudomonads on the marine alga *Saccharina latissima*. Aquat Microb Ecol 67:239–249.

20. Heiman CM, Wiese J, Kupferschmied P, Maurhofer M, Keel C, Vacheron J. 2020. Draft genome sequence of *Pseudomonas* sp. LD120 isolated from the marine alga *Saccharina latissima*. Microbiol Resour Announc 9: e01305-19.

21. Loper JE, Hassan KA, Mavrodi DV, Davis EW, Lim CK, Shaffer BT, Elbourne LDH, Stockwell VO, Hartney SL, Breakwell K, Henkels MD, Tetu SG, Rangel LI, Kidarsa TA, Wilson NL, van de Mortel JE, Song C, Blumhagen R, Radune D, Hostetler JB, Brinkac LM, Durkin AS, Kluepfel DA, Wechter WP, Anderson AJ, Kim YC, Pierson LS, Pierson EA, Lindow SE, Kobayashi DY, Raaijmakers JM, Weller DM, Thomashow LS, Allen AE, Paulsen IT. 2012. Comparative genomics of plant-associated *Pseudomonas* spp.: insights into diversity and inheritance of traits involved in multitrophic interactions. PLoS Genet 8:e1002784.

22. De Vrieze M, Pandey P, Bucheli TD, Varadarajan AR, Ahrens CH, Weisskopf L, Bailly A. 2015. Volatile organic compounds from native potato-associated *Pseudomonas* as potential anti-oomycete agents. Front Microbiol 6:1296.

23. Pierson LS, Thomashow LS. 1992. Cloning and heterologous expression of the phenazine biosynthetic locus from *Pseudomonas aureofaciens* 30-84. Mol Plant Microbe Interact 5:330–339.

24. Jiang Q, Xiao J, Zhou C, Mu Y, Xu B, He Q, Xiao M. 2014. Complete genome sequence of the plant growth-promoting rhizobacterium *Pseudomonas aurantiaca* strain JD37. J Biotechnol 192 Pt A:85–86.

25. Fang R, Lin J, Yao S, Wang Y, Wang J, Zhou C, Wang H, Xiao M. 2013. Promotion of plant growth, biological control and induced systemic resistance in maize by *Pseudomonas aurantiaca* JD37. Ann Microbiol 63:1177–1185.

26. Kluyver AJ. 1956. *Pseudomonas aureofaciens* nov. spec. and its pigments. J Bacteriol 72:406–411.

27. Peix A, Valverde A, Rivas R, Igual JM, Ramírez-Bahena M-H, Mateos PF, Santa-Regina I, Rodríguez-Barrueco C, Martínez-Molina E, Velázquez E. 2007. Reclassification of *Pseudomonas aurantiaca* as a synonym of *Pseudomonas chlororaphis* and proposal of three subspecies, *P. chlororaphis* subsp. *chlororaphis* subsp. nov., *P. chlororaphis* subsp. *aureofaciens* subsp. nov., comb. nov. and *P. chlororaphis* subsp. *aurantiaca* subsp. nov., comb. nov. Int J Syst Evol Microbiol 57:1286–1290.

28. Burr SE, Gobeli S, Kuhnert P, Goldschmidt-Clermont E, Frey J. 2010. *Pseudomonas chlororaphis* subsp. *piscium* subsp. nov., isolated from freshwater fish. Int J Syst Evol Microbiol 60:2753–2757.

29. Chin-A-Woeng TFC, Bloemberg GV, van der Bij AJ, van der Drift KMGM, Schripsema J, Kroon B, Scheffer RJ, Keel C, Bakker PAHM, Tichy H-V, de Bruijn FJ, Thomas-Oates JE, Lugtenberg BJJ. 1998. Biocontrol by phenazine-1-carboxamide-Producing *Pseudomonas chlororaphis* PCL1391 of tomato root rot caused by *Fusarium oxysporum* f. sp. *radicis-lycopersici*. Mol Plant Microbe Interact 11:1069–1077.

30. Zeng Q, Wang J, Bertels F, Giordano PR, Chilvers MI, Huntley RB, Vargas JM, Sundin GW, Jacobs JL, Yang C-H. 2017. Recombination of virulence genes in divergent *Acidovorax avenae* strains that infect a common host. Mol Plant Microbe Interact 30:813–828.

31. Reinhold B, Hurek T, Niemann E-G, Fendrik I. 1986. Close association of *Azospirillum* and diazotrophic rods with different root zones of kallar grass. Appl Environ Microbiol 52:520–526.

32. Pimentel JP, Olivares F, Pitard RM, Urquiaga S, Akiba F, Döbereiner J. 1991. Dinitrogen fixation and infection of grass leaves by *Pseudomonas rubrisubalbicans* and *Herbaspirillum seropedicae*, p. 225–229. *In* Polsinelli, M, Materassi, R, Vincenzini, M (eds.), Nitrogen Fixation: Proceedings of the Fifth International Symposium on Nitrogen Fixation with Non-Legumes, Florence, Italy, 10–14 September 1990. Springer Netherlands, Dordrecht.

33. Cava JR, Elias PM, Turowski DA, Noel KD. 1989. *Rhizobium leguminosarum* CFN42 genetic regions encoding lipopolysaccharide structures essential for complete nodule development on bean plants. J Bacteriol 171:8–15.

34. Martínez E, Palacios R, Sánchez F. 1987. Nitrogen-fixing nodules induced by *Agrobacterium tumefaciens* harboring *Rhizobium phaseoli* plasmids. J Bacteriol 169:2828–2834.

35. Kupferschmied P, Chai T, Flury P, Blom J, Smits THM, Maurhofer M, Keel C. 2016. Specific surface glycan decorations enable antimicrobial peptide resistance in plant-beneficial pseudomonads with insect-pathogenic properties. Environ Microbiol 18:4265–4281.

36. Simon R, Priefer U, Pühler A. 1983. A broad host range mobilization system for *in vivo* genetic engineering: transposon mutagenesis in gram-negative bacteria. Nat Biotechnol 1:784–791.
